# Evaluation of COVID-19 Effect on Mental Health, Self-Harm, and Suicidal Behaviors in Children and Adolescents Population

**DOI:** 10.3390/jcm13030744

**Published:** 2024-01-27

**Authors:** Jagoda Grzejszczak, Dominik Strzelecki, Agata Gabryelska, Magdalena Kotlicka-Antczak

**Affiliations:** 1 Department of Child and Adolescent Psychiatry, Medical University of Lodz, 92-216 Lodz, Poland; magdalena.kotlicka-antczak@umed.lodz.pl; 2 Department of Affective and Psychotic Disorders, Medical University of Lodz, 92-216 Lodz, Poland; dominik.strzelecki@umed.lodz.pl; 3 Department of Sleep Medicine and Metabolic Disorders, Medical University of Lodz, 92-215 Lodz, Poland; agata.gabryelska@gmail.com

**Keywords:** mental well-being, depression, anxiety, self-harm, suicidal thoughts, children, adolescents, COVID-19 pandemic

## Abstract

Objectives: The impact of the COVID-19 pandemic on the psychological state of the under-18 population includes an increased risk of psychopathological symptoms development and exacerbation of already present psychiatric disorders. This study aimed to assess the prevalence of mental health problems in Polish children and adolescents with a focus on suicidal and self-harm behavior with the impact of the pandemic. Methods: The questionnaire collected demographic data, information regarding mental states and psychopathological symptoms, history of self-harm and suicidal behaviors, as well as the experience of psychological, and physical violence, and suicidal self-harm behaviors before and during the COVID-19 pandemic. Results: In the final analysis, 782 responses were included. Self-evaluation of general and mental health scores was significantly lower during the pandemic among children (both *p* < 0.001) and adolescents (both *p* < 0.001). Moreover, general and mental health scores were lower among adolescents compared to children before (both *p* < 0.001) and during (both *p* < 0.001) the pandemic. The frequency of seeking help because of mental health problems increased during the pandemic among children and adolescents, while no changes were observed in the prevalence of psychiatric hospitalizations in either of the populations (*p* = 0.317 and *p* = 1.00, respectively). Out of autoregressive behaviors among children during the pandemic period, only the frequency of thinking about death increased (*p* = 0.038). No suicidal attempts were undertaken by children in either of the evaluated time periods. The presence of all autoaggressive behaviors was greater among adolescents compared to children both before and during the COVID-19 pandemic (all *p*<0.05). Conclusions: A subjective decrease in psychophysical well-being, an increase in the frequency of seeking mental health help during the pandemic, as well as an increased prevalence of depressive and anxiety symptoms were observed in the under-18 population as a potential consequence of the COVID-19 pandemic and related socioeconomic changes. The marked increase in self-harm behavior in the adolescent population (age > 12) and the marked increase in the frequency of death thinking in children (age ≤ 12) suggests the need for greater awareness and easier access to professional help from mental health specialists, particularly in a time of unprecedented stress and social isolation.

## 1. Introduction

The COVID-19 pandemic caused significant life changes for both adults and children, providing a vast amount of stress and uncertainty at the same time [1]. Much attention has been paid to the somatic consequences of COVID-19 infection. In most children, the COVID-19 infection was mostly asymptomatic or mild with only certain initial features increasing the risk of moderate to severe disease in selected cases [2]. While researchers point to a number of psychiatric symptoms (mood disorders, anxiety symptoms, sleep disorders) occurring during the pandemic in the under-18 population they do not provide an elucidation of the genesis of the symptoms. It is the infection itself, or rather, it is associated with a psychological response to a number of problems imposed during epidemiological restrictions [1,3,4] ? The long-term consequences of pandemic social isolation are likely to be observed for many years [5].

The unprecedented changes brought about by COVID-19, such as social isolation, school closures, and family stress, negatively affect people’s mental health, especially that of children and adolescents [6]. Most meta-analyses conducted so far have demonstrated the negative impact of the period of epidemiological isolation on mental well-being in the population under 18 years of age, including an increase in the incidence of anxiety, depression, sleep disorders, suicidal behavior, stress-related disorders, attention deficit hyperactivity disorder, and other mental problems [7,8,9,10]. Researchers have found several variables such as age, grade level, education level, gender, geographic region, and electronic use to be correlated with the incidence of mental health symptoms [11]. American researchers have also reviewed circadian rhythm disorders in the pediatric population, pointing out that in crisis situations, parameters such as extended sleep time, sleep latency, or wake-up time change, at the same time mentioning that sleep may play a therapeutic role in a previously unknown mechanism [12,13]. The concept of loneliness seems to be an important issue in the pediatric population; reviews indicate that it is an important risk factor for the severity of psychopathological symptoms in this group in the era of epidemiological isolation [14]. Reports from Israel raise alarm about cognitive disorders occurring in children during the pandemic, which not only represent a somatic complication but are also related to the economic and emotional stress of parents [15]. Few studies indicate the possibility of correlating psychopathological symptoms with possible post-traumatic stress symptoms, indicating the strength of the pandemic stressor in children and adolescents [16].

The spectrum of suicide and self-harm behavior remains an extremely important issue in the context of the COVID-19 pandemic in people under 18 years of age [17]. Research results on this topic are inconsistent. However, most of them mention the occurrence of suicidal thoughts themselves and not the frequency of suicidal behavior [18]. By contrast, the increase in the occurrence of self-harm behavior intensity has been noted in most studies [19]. All reports emphasize preventive actions related to life-protective factors [20].

A clearly greater susceptibility to mental health problems was mentioned in children and adolescents with significant psychological or psychiatric problems at the beginning, again emphasizing the importance of support strategies [21]. The important role of educational institutions as a support tool in the crisis was also emphasized [22]. Moreover, the summaries made so far regarding the disturbance of mental well-being emphasize protective factors, such as strong and supportive home and peer relationships, as well as the importance of preventive actions and the development of support strategies for children affected by the crisis [9,23,24]. Reports from Montreal show the work–home correlation of parents of children with mental problems, suggesting that the workload and lack of favorable working conditions of a parent during the pandemic worsen the child’s mental well-being [25].

Most of the available studies conducted on the Polish population during the period of our research concern the population of adults from various age ranges and social groups (medical students, nurses, patients with chronic diseases, pregnant women, and people from the geriatric population) [26,27,28,29]. Available research from the period of the COVID-19 pandemic regarding its impact on the mental well-being of the Polish population under 18 years of age mainly concerns teenagers and focuses on the severity of depression and anxiety symptoms [30]. Colleagues from our university, while examining the neuropsychiatric complications of the virus infection itself, also mention cognitive dysfunctions—significant difficulties with concentration and general memory loss—also pointing out that the isolation itself during the pandemic could be a strong enough stressor to cause symptoms of post-traumatic stress disorder [31]. The role of physical activity as a protective factor against the deterioration of mental health in the adolescent population has also been emphasized [32,33]. Colleagues from the center in Bydgoszcz attempted to assess the long-term consequences of social isolation in correlation with the armed conflict in Ukraine but showed that the severity of depressive and anxiety symptoms was moderate [34]. The few studies on self-aggressive behavior, including suicidal behavior, in the context of the COVID-19 pandemic in Poland indicate the intensification of the phenomenon mainly in adolescents previously undergoing psychiatric treatment, at the same time emphasizing the importance of preventive measures and interventions by pediatricians [35,36].

Many review articles covering existing research point to the inconsistency of reports, pointing to the lack of a clear narrowing of the population, the fact that the tools used were not adapted to the specific conditions of the pandemic, and the lack of direct measurement during the crisis as weak points [37,38]. Thus, the study aimed to assess the prevalence of mental health problems in Polish children and adolescents with a focus on suicidal and self-harm behavior and the frequency of reported symptoms.

## 2. Materials and Methods

### 2.1. Study Design and Sample

The study included the population of children and adolescents of Lodz Voivodeship, Poland, during the second (2021) and the fourth wave (2022) of the COVID-19 pandemic. The age of the respondents covered the population range of 6–18 years, which is the age of compulsory education in Poland. The self-administered anonymous online questionnaire available on an online Google Excel spreadsheet was used to assess the psychological state of children and adolescents in the age range of 6–18 years, which is the age of compulsory education in Poland. Participants were invited to take the survey through individual contact with the directors of primary and secondary schools engaged in the education of children aged 6–18 years in the Lodz area. Of the 3383 registered institutions of this type, 86 schools were invited to the survey. Facilities intended for students under 6 years of age were not invited to participate in the study. Some schools did not respond to the invitation or directly refused to participate. Each principal received the content of the questionnaire for review and discussion with the school’s psychological and pedagogical team before the questionnaires were distributed to parents via e-mail. Those parents, after agreeing to their child’s participation in the study, passed them on to their children. Study participants were guaranteed confidentiality and anonymity, and no data that could help to identify a responder were collected. The study was conducted in accordance with the Helsinki Declaration. Participation in the study was voluntary. All participants were informed about the study design and provided informed consent to participate. Participation could have been withdrawn at any time during the course of the survey. 

### 2.2. Measurement Tools

The survey was prepared using Google Forms (see Supplementary Materials for Polish language versions of the questionnaire). The introductory part of the questionnaire included demographic data regarding the participants, their household, and their family members. The main part of the questionnaire referred to two different time points: first, during the pandemic (present moment while filling out the questionnaire), and second, before the pandemic (retrospectively). General and mental health were evaluated by participants on a Likert-like scale of 1 to 10 (where 1 indicates significant health problems and 10 indicates full health well-being). It was followed by questions covering psychopathological symptoms and mental problems, such as the presence of depressive symptoms, anxiety, psychotic symptoms, and tics as well as abuse of psychoactive substances and the need for specialist consultation or hospitalization, which were answered in “yes or no” format. Autoaggressive behaviors were similarly assessed with “yes or no”; self-harm, suicidal thoughts, and suicidal tendencies were answered in a “yes or no” manner with a follow-up regarding their frequency in a Likert-like scale from 1 to 5 gradually (where 1 means not present and means 5 very often). In the next part, the subjective level of intensity of chosen symptoms (such as sense of meaning, level of worry, sense of overload, nervousness, irritability, confusion, sense of lack of value, anhedonia, anergy, sleep disorders, loneliness, and crying) was assessed with a Likert-like scale from 1 to 5 gradually (where 1 means no symptom; 5 means very strong intensity). A subsection regarding questions about physical violence, psychological violence, and physical activity was described and analyzed in a separate study [39]. In addition, the prevalence of COVID-19 infection concerns, the need to maintain social contacts during the pandemic, and performance at school were assessed. The questionnaire included questions about physical violence, psychological violence, and physical activity. The chapters and sections of the questionnaire are graphically presented in Table 1. In the analysis of the results, these 2 time points were referred to as during the pandemic and before the pandemic/pre-pandemic period.

### 2.3. Statistical Analysis

Statistical analysis was performed at a significance level of 0.05 using two-tailed tests. The normality of the distribution of variables was tested with the Shapiro–Wilk test. For ordinal variables, the data are shown as the number of cases and the percentage of the full group; for variables with a distribution other than normal, the data are presented as the median with the interquartile range (IQR). Chi-square, Chi-square tests with Yate’s correction, and Fisher’s test were used to assess nominal variables in situations in which the size of the smallest group was, respectively, above 15, in the range of 5–15, and below 5. Comparisons of independent groups were made using the Mann–Whitney U test (for variables with a different distribution than normal) (analysis between groups at the same time point). Dependent groups were compared with Wilcoxon (for variables with a different distribution than normal) (analysis between two time points in the same group). The analysis was performed using SPSS Statistics version 28 (IBM, Armonk, NY, USA).

## 3. Results

### 3.1. Group Characterization

Eight hundred twenty-three responses were collected. From the analysis, 41 individuals were excluded (1 due to a wrong date of birth and 40 due to not fulfilling the age criteria for inclusion—over 18 years old). Furthermore, since only 2 participants responded that they lived during the pandemic period in the capital, Warsaw, they were added to the group who lived in a city of 100–1000 thousand together, in effect making a group living in a city with over 100 thousand population. For purposes of the analysis, two age-based subgroups were created: children (age ≤ 12) and adolescents (age > 12). In the final analysis, 782 responses were included; 480 responses were collected during the second wave of COVID-19, and 302 were collected during the fourth wave. Out of all participants, 109 were children (age ≤ 12), while 673 individuals belonged to the adolescent group (age > 12). The general characteristics of the participants are presented in Table 2.

### 3.2. General and Mental Health Assessment

Self-evaluation of both general and mental health was worse during the pandemic among children and adolescents (both *p* < 0.001). Moreover, general and mental health scores were lower among adolescents compared to children before and during the pandemic (both *p* < 0.001). The frequency of seeking help from mental health services increased during the pandemic among children and adolescents, while no changes were observed in the prevalence of psychiatric hospitalizations in either of the populations (*p* = 0.317 and *p* = 1.00, respectively). The prevalence of depression and anxiety increased during the pandemic compared to the pre-pandemic period in children and adolescents (all *p* < 0.001); furthermore, the reported frequency of both disorders was greater among adolescents compared to children before and during the COVID-19 pandemic (for all variables, *p* < 0.001). Full data on general and mental health self-evaluation, frequency of psychiatric diagnoses, and usage of help from mental health specialists are presented in Table 3.

No statistically significant differences were observed between children and adolescents regarding changes in the general health evaluation, while 9.2% and 18.4% of individuals, respectively, reported the deterioration of their general health. On the other hand, 11.9% of children and 15.9% of adolescents stated a decline in their mental health during the pandemic compared to the pre-pandemic period (*p* = 0.049). The summary of changes in general and mental health are shown in Table 4.

### 3.3. Evaluation of Symptom Prevalence

The frequency of all symptoms reported by participants increased during COVID-19 compared to the pre-pandemic period among both children and adolescents (all *p* < 0.001); furthermore, the frequency of all symptoms was greater among adolescents compared to children both before and during the pandemic (all *p* < 0.001). Among children, the frequency of mental problems increased the most from before to during the pandemic period followed by nervousness, irritability, and worrying, while adolescents reported the greatest increase in worrying about the future during the COVID-19 pandemic compared to the pre-pandemic period, followed by confusion and anergy. A summary of the frequency of mental health-related symptoms before and during the COVID-19 pandemic is presented in Table 5.

### 3.4. Autoagressive Behaviors

Out of autoregressive and suicidal behaviors among children, only the frequency of thinking about death increased during the pandemic period (*p* = 0.038). In this group, only 1 out of 109 participants reported self-harm both before and during the pandemic; the presence of suicidal thoughts was stated by 5 and 6 individuals before and during the pandemic respectively. No suicidal attempts were undertaken by children in either of the evaluated time periods. Among adolescents, no changes between before and during the pandemic period were observed regarding the presence of suicidal thoughts (36.6% vs. 38.0%; *p* = 0.307) and suicidal attempts (5.8% vs. 4.9%; *p* = 0.317). Furthermore, the frequency of suicidal thoughts and thoughts about death increased during the pandemic (both *p* = 0.007). Adolescents reported undertaking self-harm more often during the pandemic than before (4.6% vs. 12.2%; *p* = 0.049), with tension reduction being the most common reason given at both time periods (85.4% and 72.0%, respectively). The presence of all autoregressive and suicidal behaviors was greater among adolescents compared to children both before and during the COVID-19 pandemic (all *p* < 0.05). Data regarding the presentation of autoaggressive/suicidal behaviors before and during the COVID-19 pandemic are shown in Table 6.

## 4. Discussion

The COVID-19 pandemic has led to disruptions in family and social life around the world due to social isolation, school closures, and growing concerns about health and material existence [24]. Although the long-term consequences of epidemiological prevention activities are not yet known, there are many studies documenting a clear impact on the mental well-being of children and adolescents under 18 years of age [5]. Many of the studies reporting the overall mental state in society have focused on children and adolescents, particularly with regard to increased emergency department visits and suicide attempts [40].

In our project, we investigated parameters of psychological well-being in the population of Lodz voivodship aged up to 18 years before and during the COVID-19 pandemic. The questions contained in the survey concerned both the current situation and, retrospectively, the situation preceding the pandemic; this is a limitation of the study, as the data from before the pandemic were not collected at that time. However, the inclusion of questions about both the period preceding and the period of the pandemic gave an overview of the emotions, mental state, and situation of children and adolescents during this crisis period. The pandemic period was inextricably linked to home isolation and thus remote learning in many Polish schools but also more frequent and prolonged contact with family members and other household members, which could either be a protective factor or cause psychopathological symptoms [41].

The process of growing up and, therefore, belonging to a group of children or adolescents has a clear impact on the frequency of occurrence of certain psychopathological symptoms; therefore, we decided to analyze the results we collected in two subgroups, age ≤ 12 and age > 12, to better analyze mental health problems by age group [42].

No study has shown the positive or lack of impact of the COVID-19 crisis on the general and mental health of the pediatric population [43]. The research conducted so far clearly shows an increase in the frequency of children seeking psychological support [44]. However, Boston researchers have shown that in the long run, in younger patients, the help initiative from adults is more important, and their cognitive flexibility and educational involvement lead to a reduced risk of symptoms of depression and anxiety in children [45]. In the pediatric population data we received, we did not note any change in the frequency of admissions due to new psychiatric problems or the exacerbation of existing ones. Contrary to global reports, researchers from Tuscany recorded a 200% increase in hospital admissions due to newly occurring psychiatric problems [45]. One possible explanation might be the difference in access to professional mental health help in different regions, both before and during the pandemic, including some limitations to hospital admittance due to epidemiological restrictions. Generally, an increasing number of studies have reported higher levels of anxiety, depression, and stress among children (ages 6–12 years) who experienced family isolation and school closures during COVID-19, which is also proven by our results [46]. Attention is also drawn to the strong sense of loneliness associated with social isolation among children, which intensifies depressive and anxiety symptoms [14].

Among self-aggressive behaviors in the children’s population, we only noted an increase in the frequency of thinking about death; none of the surveyed children attempted suicide or showed such tendencies. Global reports in this area are inconsistent and indicate schools as potential sources of mental psychopathology symptoms. For example, researchers from Alicante reported a significant increase in both suicidal thoughts and suicide attempts among children [47]. In turn, Welsh research mentioned a constant upward trend in this area, not strictly related to the COVID-19 pandemic crisis [48]. Researchers point to a correlation between mental disorders that lead to suicidal thoughts and tendencies in children. It should be noted that in our study population <12 years of age, no mental disorders were noticed among the respondents, which may explain the milder consequences in the form of suicidal thoughts only without self-aggressive behavior [49].

Research on the psychophysical condition of teenagers has also confirmed the lack of well-being and psychological consequences in this age group—depressive disorders, anxiety, and sleep problems are just some of those caused by pandemics [50].

Researchers have pointed out how important the role of parents is, as they are a stable base in not only a teenager’s crisis but also a situational crisis such as a pandemic [51,52]. While the research studies available so far indicate a clear increase in the frequency of seeking help in the field of mental health among adolescents, some of them indicate a delayed availability of help due to epidemiological restrictions [53,54]. This is an important point for developing aid strategies for the future so that no one is left without help [55]. Some researchers have pointed out the significant role of social media in helping teenagers in times of crisis through quick availability and wider access to a dedicated support group [56,57]. We can use this for psychoeducational and support purposes. Global research has also confirmed the increase in the number of hospitalizations of adolescents during the COVID-19 pandemic, mentioning that they most often concern teenagers with eating disorders and self-harm [58,59,60].

Global reports, as well as the results of our research, indicate that the main problem that has become more intense is self-aggressive behavior [61,62]. Koreans reported that girls were much more likely to seek help in this area [63]. There is also an increase in the frequency of thinking about death and the occurrence of suicidal thoughts. The greatest intensity was observed in self-harm for the purpose of reducing tension, which is in line with the results obtained in our study [64]. At the same time, self-harm in Canada during the COVID-19 pandemic was the fourth cause of hospital admissions among teenagers [65]. Moreover, a publication by scientists from Louisiana indicated a clear correlation between engaging in suicidal behavior and the experience of bullying at school in the adolescent population [66]. Teenagers experiencing peer violence are more likely to think about death or engage in suicidal behavior [67]. The problem seems to be more severe in developing countries such as Liberia, Chile, and Indonesia [68,69,70]. Potentially limiting peer contacts may be a protective factor in engaging in suicidal Potentially limiting peer contacts may be a protective factor in engaging in suicidal behavior. Similarly, in the pediatric population, a constant upward trend in self-aggressive behavior similar to the abovementioned trends in suicidal thoughts and attempts that have been occurring for several years, unrelated to the pandemic period itself, is emphasized [71].

Other psychiatric symptoms occurring in people under 18 years of age, such as psychosis or tics, as well as the use of psychoactive substances, are also important. Canadian reports show a 66% increase in the incidence of psychosis during a pandemic in the adolescent population, which was not proven by our research [72]. Our results also did not show that the severity of tics during isolation was related to the COVID-19 pandemic; meanwhile, Danish researchers indicated that in 40% of the population of teenagers diagnosed with tics they studied, the trigger to recurrence or exacerbation was epidemiological isolation [73].

Analyzing our results, we can notice a slight increase in the use of psychoactive substances only in the adolescent population, which is consistent with global reports. American researchers showed an increase in the use of ethanol, cannabinoids, and opiates related to the immediate outbreak of the pandemic [74]. A slightly different effect was shown by researchers from Iceland, who demonstrated an increase in recreational alcohol consumption related to the loosening of pandemic restrictions and the possibility of renewed social contacts without restrictions [75]. In Poland, many places selling alcohol were closed for a long time. A clear increase in externalizing behaviors (self-harm or the use of psychoactive substances), especially in the adolescent population, proves the reactive nature of the above symptoms, thus indicating a special area for preventive actions.

It is undeniable that the pandemic has impacted the physical and mental well-being of the under-18 population. It is important to pay attention to the dynamics of problems and appropriate preventive actions, as well as help in difficult situations as quickly as possible, in order to prevent the long-term consequences of similar global crises in the future.

It is important to mention the limitations of the presented research. Because the survey was conducted online, it is difficult to verify the respondents and their affiliation with the target group. On the other hand, the form was forwarded directly to parents of students from specific educational institutions, which limits the aforementioned inadequacy of responses. Direct access to the questionnaire by parents could theoretically limit access to the questionnaire by children from violent families due to the deliberate concealment of the phenomenon occurring at home, despite the anonymity of the questionnaire. The age of the studied population covered a relatively wide range, which can be considered both an advantage and a limitation of the study. The degree of understanding and interpretation of the questions included in the questionnaire might have been different depending on the age of responders and limited especially in younger children. Due to the design of the study itself, it is burdened, as it is not a prospective observational study. The partially retrospective character of the study can also be considered a limitation. Participants during the pandemic answered questions regarding the present time (COVID-19 pandemic) and were asked to assess the situation before the pandemic retrospectively. Therefore, the retrospective answers could have been influenced by the time that passed from individuals’ experiences; the answers could have been diminished or exaggerated, and thus the results obtained in the study should be interpreted with caution, taking into consideration this limitation of the study design. 

Unfortunately, very little has been publicized about children between birth and 5 years of age, who may be among the most vulnerable to the psychosocial impacts of COVID-19 [76]. Our research was conducted in the Polish student population, so it also does not cover children under 6 years of age.

Despite reports from many countries around the world, it seems reasonable to collect data locally, as occurred in our study. This makes it possible to analyze the cultural context and the specific functioning of family systems. This allows for a more accurate assessment of needs and the optimal adjustment of preventive interventions.

## 5. Conclusions

The COVID-19 pandemic can be considered a health, economic, and social crisis. The subjective decrease in psychophysical well-being, an increase in the frequency of seeking mental health help during the pandemic, as well as an increased prevalence of depressive and anxiety symptoms were observed in the under-18 population as a potential consequence of the COVID-19 pandemic. A marked increase in self-harm behavior in the adolescent population (age > 12) and a marked increase in the frequency of death thinking in children (age ≤ 12) were observed. In parallel, a slight increase in the use of psychoactive substances during the pandemic in the youth population was demonstrated, and no change in the impact of the pandemic on psychotic disorders or tics was noted in the population of children and adolescents. At the same time, this unprecedented situation provided an opportunity to work out and optimize both preventive and recovery strategies. As far as such strategies targeted at the population under 18 years are considered, research conducted so far emphasizes the importance of supportive family and peer relationships as well as the need for the availability of professionals in the field of mental health [56,77]. Adjusting the kind of help provided to the specific local circumstances and needs should also be taken into consideration. We believe that the results of the study will allow decision-makers to pay more attention to the issue of mental health in children and adolescents and to improve forms of prevention targeting reducing the impact of the crisis situation on the everyday well-being of Polish students. The obtained data clearly indicate the importance of providing prompt help to children and adolescents struggling with suicidal thoughts as prevention of suicidal behaviors and other mental problems, as well as the need for access to the management of self-harm behaviors triggered by a crisis situation. Psychoeducation and methods for dealing with them should also be an essential element of preventive and recovery programs, the need for which is highlighted by the growing trends in the occurrence of suicidal thoughts and self-aggression, regardless of the pandemic.

## Figures and Tables

**Table 1:** Chapters and sections of the questionnaire used.

Introductory Part	Main Part	Additional Section
demographic data	depressive symptoms	physical violence
household	anxiety	psychological violence
family members	psychotic symptoms	physical activity
general well-being	tics	
mental state	use of psychoactive substances	
COVID-19 infection concerns	self-harm	
need to maintain social contacts	suicidal thoughts	
coping at school	suicidal tendencies	
	sense of meaning	
	level of worry	
	sense of overload	
	nervousness	
	irritability	
	confusion	
	sense of lack of value	
	anhedonia	
	anergy	
	sleep disorders	
	loneliness	
	crying	

**Table 2:** General characterization of study groups with comparisons of children and adolescents.

		Whole Group	Children (Age ≤ 12)	Adolescents (Age > 12)	*p*-Value
Group Size		782	109	673	N/A
Wave	Second	61.4%	100.0%	55.1%	<0.001
	Fourth	38.6%	0.0%	44.9%	
Sex	Man	37.5%	48.6%	35.7%	0.029
	Woman	62.1%	51.4%	63.9%	
	Non-binary	0.4%	0.0%	0.4%	
Education level	Primary school	47.4%	100.0%	39.1%	<0.001
	Secondary school	42.6%	0.0%	49.5%	
	Profiled high school	2.4%	0.0%	2.8%	
	Technical school	6.9%	0.0%	8.0%	
	Vocational school	0.6%	0.0%	0.6%	
Residence (number of inhabitants)	City, more than 100 thousand	8.2%	12.8%	7.4%	0.004
	City, 20–100 thousand	30.8%	30.3%	30.9%	
	Town, 10–20 thousand	10.7%	21.1%	9.1%	
	Town, < 10 thousand	4.9%	6.4%	4.6%	
	Village	45.4%	29.4%	48.0%	
Age (years old)		15.00 (13.00–17.00)	11.00 (9.00–12.00)	16.00 (14.00–17.00)	<0.001

Data presented as number of cases (%) or median (interquartile range).

**Table 3:** Comparisons of general and mental health self-evaluation, frequency of psychiatric diagnoses, and usage of help from mental health specialists.

		Full Group			Children (Age ≤ 12)			Adolescents (Age > 12)			*p*-Value (Before the Pandemic Children vs. Adolescents)	*p*-Value (during the Pandemic Children vs. Adolescents)
		Before the Pandemic	During the Pandemic	*p*-Value	Before the Pandemic	During the Pandemic	*p*-Value	Before the Pandemic	During the Pandemic	*p*-Value		
		n = 782			n = 109		n = 673					
General health		9.00 (7.00–10.00)	8.00 (6.00–9.00)	<0.001	9.00 (8.00–10.00)	8.00 (7.00–10.00)	<0.001	8.00 (7.00–9.00)	8.00 (6.00–9.00)	<0.001	<0.001	<0.001
Mental health		8.00 (7.00–10.00)	7.00 (4.00–9.00)	<0.001	10.00 (9.00–10.00)	8.00 (6.00–9.00)	<0.001	8.00 (7.00–9.00)	6.00 (4.00–8.00)	<0.001	<0.001	<0.001
Mental health problems	No	79.4%	67.5%	<0.001	100.0%	88.1%	<0.001	76.1%	64.2%	<0.001	<0.001	<0.001
	Yes	20.6%	32.5%		0.0%	11.9%		23.9%	35.8%			
Use of consultation from specialists (psychologist, psychotherapist, psychiatrist)	No	88.7%	84.4%	<0.001	99.1%	90.8%	0.007	87.1%	83.4%	0.003	<0.001	0.046
	Yes	11.3%	15.6%		0.9%	9.2%		12.9%	16.6%			
Psychiatric hospitalization	No	97.6%	97.4%	0.835	100.0%	99.1%	0.317	97.2%	97.2%	1.000	0.076	0.243
	Yes	2.4%	2.6%		0.0%	0.9%		2.8%	2.8%			
Depression	No	91.9%	82.0%	<0.001	100.0%	94.5%	0.014	90.6%	79.9%	<0.001	<0.001	<0.001
	Yes	8.1%	18.0%		0.0%	5.5%		9.4%	20.1%			
Anxiety	No	88.7%	81.8%	<0.001	100.0%	93.6%	0.008	86.9%	79.9%	<0.001	<0.001	<0.001
	Yes	11.3%	17.9%		0.0%	6.4%		13.0%	20.1%			
Psychosis	No	98.7%	98.5%	0.480	100.0%	100.0%	1.000	98.5%	98.2%	0.480	0.201	0.160
	Yes	1.3%	1.5%		0.0%	0.0%		1.5%	1.8%			
Tics	No	96.3%	96.5%	0.670	100.0%	100.0%	1.000	95.7%	96.0%	0.670	0.027	0.033
	Yes	3.7%	3.5%		0.0%	0.0%		4.3%	4.0%			
Use of psychoactive substances	No	99.4%	97.8%	0.004	100.0%	100.0%	1.000	99.3%	97.5%	0.005	0.367	0.094
	Yes	0.6%	2.2%		0.0%	0.0%		0.7%	2.5%			

Data presented as number of cases (%) or median (interquartile range).

**Table 4:** Changes in general and mental health from before to during the COVID-19 period.

		Full Group	Children (Age ≤ 12)	Adolescents (Age > 12)	*p*-Value
General health (change from pre-COVID-19 to during COVID-19)	deterioration	134 (17.1%)	10 (9.2%)	124 (18.4%)	0.053
	without changes	262 (33.5%)	42 (38.5%)	220 (32.7%)	
	improvement	386 (49.4%)	57 (52.3%)	329 (48.9%)	
Mental health condition (change from pre-COVID-19 to during COVID-19)	deterioration	120 (15.3%)	13 (11.9%)	107 (15.9%)	0.049
	without changes	635 (81.2%)	96 (88.1%)	539 (80.1%)	
	improvement	27 (3.5%)	0 (0.0%)	27 (4.0%)	

Data presented as number of cases (%).

**Table 5:** Frequency of mental health-related symptoms before and during COVID-19 pandemic.

Symptom	Frequency of Symptom Presence	Full Group			Children (Age ≤ 12)			Adolescents (Age > 12)			*p*-Value (Before the Pandemic Children vs. Adolescents)	*p*-Value (During the Pandemic Children vs. Adolescents)
		Before the Pandemic	During the Pandemic	*p*-Value	Before the Pandemic	During the Pandemic	*p*-Value	Before the Pandemic	During the Pandemic	*p*-Value		
		n = 782			n = 109			n = 673				
Sense of security	1	32.4%	17.4%	<0.001	48.6%	20.2%	<0.001	29.7%	16.9%	<0.001	<0.001	0.025
	2	23.7%	22.0%		22.9%	28.4%		23.8%	21.0%			
	3	26.0%	36.2%		15.6%	33.9%		27.6%	36.6%			
	4	12.1%	17.0%		6.4%	13.8%		13.1%	17.5%			
	5	46 (5.9%)	7.4%		6.4%	4 (3.7%)		5.8%	8.0%			
Worries about the future	1	37.0%	19.1%	<0.001	52.3%	20.2%	<0.001	34.5%	18.9%	<0.001	<0.001	0.003
	2	24.3%	17.1%		22.9%	25.7%		24.5%	15.8%			
	3	23.9%	29.9%		16.5%	34.9%		25.1%	29.1%			
	4	10.4%	22.0%		7.3%	14.7%		10.8%	23.2%			
	5	4.5%	11.9%		0.9%	4.6%		5.1%	13.1%			
Feeling overwhelmed	1	24.8%	19.7%	<0.001	49.5%	28.4%	<0.001	20.8%	18.3%	<0.001	<0.001	<0.001
	2	19.4%	16.1%		18.3%	18.3%		19.6%	15.8%			
	3	28.1%	25.7%		20.2%	30.3%		29.4%	25.0%			
	4	16.8%	21.4%		9.2%	16.5%		18.0%	22.1%			
	5	10.9%	17.1%		2.8%	6.4%		12.2%	18.9%			
Nervousness	1	21.6%	14.8%	<0.001	41.3%	17.4%	<0.001	18.4%	14.4%	<0.001	<0.001	0.024
	2	21.0%	17.8%		23.9%	20.2%		20.5%	17.4%			
	3	25.6%	24.7%		18.3%	26.6%		26.7%	24.4%			
	4	19.4%	22.5%		10.1%	27.5%		21.0%	21.7%			
	5	12.4%	20.2%		6.4%	8.3%		13.4%	22.1%			
Irritability	1	24.3%	19.6%	<0.001	36.7%	22.0%	<0.001	22.3%	19.2%	<0.001	<0.001	0.026
	2	21.5%	14.6%		26.6%	18.3%		20.7%	14.0%			
	3	26.3%	23.7%		19.3%	22.9%		27.5%	23.8%			
	4	15.0%	18.8%		10.1%	26.6%		15.8%	17.5%			
	5	12.9%	23.4%		7.3%	10.1%		13.8%	25.6%			
Worrying	1	17.5%	16.2%	<0.001	32.1%	23.9%	<0.001	15.2%	15.0%	<0.001	<0.001	0.003
	2	21.1%	17.8%		28.4%	16.5)		19.9%	18.0%			
	3	26.0%	22.4%		23.9%	24.8%		26.3%	22.0%			
	4	20.1%	21.1%		11.0%	25.7%		21.5%	20.4%			
	5	15.3%	22.5%		4.6%	9.2%		17.1%	24.7%			
Confusion	1	25.2%	20.3%	<0.001	38.5%	22.0%	<0.001	23.0%	20.1%	<0.001	<0.001	0.012
	2	20.5%	15.1%		30.3%	21.1%		18.9%	14.1%			
	3	27.2%	21.1%		19.3%	22.9%		28.5%	20.8%			
	4	15.1%	22.6%		8.3%	24.8%		16.2%	22.3%			
	5	12.0%	20.8%		3.7%	9.2%		13.4%	22.7%			
Worthlessness	1	41.9%	39.9%	<0.001	67.9%	63.3%	0.074	37.7%	36.1%	<0.001	<0.001	<0.001
	2	18.9%	13.8%		14.7%	13.8%		19.6%	13.8%			
	3	18.2%	17.8%		12.8%	15.6%		19.0%	18.1%			
	4	12.7%	13.8%		1.8%	4.6%		14.4%	15.3%			
	5	8.3%	14.7%		2.8%	2.8%		9.2%	16.6%			
No prospects	1	37.6%	36.6%	<0.001	58.7%	59.6%	0.290	34.2%	32.8%	<0.001	<0.001	<0.001
	2	21.0%	16.0%		22.9%	15.6%		20.7%	16.0%			
	3	18.8%	18.5%		11.9%	15.6%		19.9%	19.0%			
	4	12.7%	13.6%		4.6%	6.4%		14.0%	14.7%			
	5	10.0%	15.3%		1.8%	2.8%		11.3%	17.4%			
Lack of sense	1	43.5%	39.1%	<0.001	67.9%	60.6%	0.007	39.5%	35.7%	<0.001	<0.001	<0.001
	2	16.9%	12.0%		14.7%	13 (11.9%)		17.2%	12.0%			
	3	17.1%	16.0%		11.0%	14.7%		18.1%	16.2%			
	4	9.5%	13.9%		4.6%	10.1%		10.3%	14.6%			
	5	13.0%	18.9%		1.8%	2.8%		14.9%	21.5%			
Decreased appetite	1	53.6%	48.6%	<0.001	72.5%	65.1%	0.013	50.5%	45.9%	<0.001	<0.001	<0.001
	2	14.6%	13.2%		13.8%	17.4%		14.7%	12.5%			
	3	17.3%	16.0%		8.3%	6.4%		18.7%	17.5%			
	4	7.7%	10.4%		3.7%	7.3%		8.3%	10.8%			
	5	6.9%	11.9%		1.8%	3.7%		7.7%	13.2%			
Anergy	1	33.1%	27.5%	<0.001	64.2%	45.9%	<0.001	28.1%	24.5%	<0.001	<0.001	<0.001
	2	20.1%	13.8%		15.6%	16.5%		20.8%	13.4%			
	3	20.7%	18.3%		7.3%	17.4%		22.9%	18.4%			
	4	13.7%	18.4%		6.4%	12.8%		14.9%	19.3%			
	5	12.4%	22.0%		6.4%	7.3%		13.4%	24.4%			
Anhedonia	1	46.9%	37.2%	<0.001	66.1%	56.9%	0.002	43.8%	34.0%	<0.001	<0.001	<0.001
	2	15.2%	13.4%		14.7%	12.8%		15.3%	13.5%			
	3	20.1%	17.9%		10.1%	13.8%		21.7%	18.6%			
	4	9.2%	15.1%		5.5%	11.0%		9.8%	15.8%			
	5	8.6%	16.4%		3.7%	5.5%		9.4%	18.1%			
Disturbed sleep	1	45.5%	40.2%	<0.001	70.6%	60.6%	<0.001	41.5%	36.8%	<0.001	<0.001	<0.001
	2	15.9%	15.3%		13.8%	11.9%		16.2%	15.9%			
	3	16.9%	16.0%		6.4%	14.7%		18.6%	16.2%			
	4	10.5%	11.1%		6.4%	6.4%		11.1%	11.9%			
	5	11.3%	17.4%		2.8%	6.4%		12.6%	19.2%			
Loneliness	1	36.8%	31.1%	<0.001	61.5%	46.8%	<0.001	32.8%	28.5%	<0.001	<0.001	<0.001
	2	18.0%	12.9%		16.5%	11.9%		18.3%	13.1%			
	3	19.3%	16.5%		12.8%	16.5%		20.4%	16.5%			
	4	13.2%	16.5%		3.7%	14.7%		14.7%	16.8%			
	5	12.7%	23.0%		5.5%	10.1%		13.8%	25.1%			
Tearfulness	1	43.5%	35.7%	<0.001	67.0%	51.4%	<0.001	39.7%	33.1%	<0.001	<0.001	<0.001
	2	15.0%	15.7%		14.7%	22.9%		15.0%	14.6%			
	3	17.8%	15.7%		8.3%	11.0%		19.3%	16.5%			
	4	11.0%	13.3%		8.3%	8.3%		11.4%	14.1%			
	5	12.8%	19.6%		1.8%	6.4%		14.6%	21.7%			

Data presented as number of cases (%).

**Table 6:** Presentation of autoaggressive and suicidal behaviors before and during the COVID-19 pandemic.

		Full Group			Children (Age ≤ 12)			Adolescents (Age > 12)			*p*-Value (Before the Pandemic Children vs. Adolescents)	*p*-Value (During the Pandemic Children vs. Adolescents)
		Before the Pandemic	During the Pandemic	*p*-Value	Before the Pandemic	During the Pandemic	*p*-Value	Before the Pandemic	During the Pandemic	*p*-Value		
		n = 782			n = 109		n = 673					
Self-harm	No	89.4%	87.5%	0.049	99.1%	99.1%	1.000	87.8%	85.4%	0.049	<0.001	<0.001
	Yes	10.6%	12.5%		0.9%	0.9%		12.2%	14.6%			
Self-harm (cause)	Tension reduction	78.3%	86.7%	0.005	100.0%	100.0%	1.000	72.0%	85.7%	0.005	0.003	<0.001
	Joy, euphoria	22.9%	15.3%	0.317	100.0%	100.0%	1.000	22.0%	14.3%	0.317	0.269	0.423
	Way to deal with boredom	15.7%	19.4%	0.180	0.0%	0.0%	1.000	15.9%	19.4%	0.180	0.114	0.076
	Way to die	18.1%	26.5%	0.005	0.0%	0.0%	1.000	18.3%	26.5%	0.005	0.116	0.037
Frequency of thoughts about death	1	58.1%	55.1%	0.002	89.9%	82.6%	0.038	52.9%	50.7%	0.007	<0.001	<0.001
	2	14.1%	13.6%		6.4%	11.0%		15.3%	14.0%			
	3	13.3%	13.0%		1.8%	3.7%		15.2%	14.0%			
	4	8.4%	11.0%		0.9%	1.8%		9.7%	12.5%			
	5	6.1%	7.3%		0.9%	0.9%		7.0%	8.3%			
Suicidal thoughts	No	67.9%	66.5%	0.269	95.4%	94.5%	0.564	63.4%	62.0%	0.307	<0.001	<0.001
	Yes	32.1%	33.5%		4.6%	5.5%		36.6%	38.0%			
Frequency of suicidal thoughts	1	67.9%	66.5%	0.031	95.4%	94.5%	0.414	63.4%	62.0%	0.007	<0.001	<0.001
	2	12.4%	9.3%		2.8%	2.8%		14.0%	10.4%			
	3	9.7%	9.2%		0.9%	1.8%		11.1%	10.4%			
	4	5.9%	8.1%		0.9%	0.9%		6.7%	9.2%			
	5	4.1%	6.9%		0.0%	0.0%		4.8%	8.0%			
Suicidal attempt	No	95.0%	95.8%	0.017	100.0%	100.0%	1.000	94.2%	95.1%	0.317	0.010	0.018
	Yes	5.0%	4.2%		0.0%	0.0%		5.8%	4.9%			

Data presented as number of cases (%). Self-harm (cause) reported as percentages of each reason referring to participants who answered yes out of those who reported self-harm behaviors at given time point.

## Data Availability

Data will be made available upon request.

## Supplementary Materials

The following supporting information can be downloaded at: https://www.mdpi.com/article/10.3390/jcm13030744/s1.
