# Supplementary material for: Evaluation of COVID-19 Effect on Mental Health, Self-Harm, and Suicidal Behaviors in Children and Adolescents Population
Source: J Clin Med. 2024 Jan 27;13(3):744. doi: 10.3390/jcm13030744 (PMC10856233; doi:10.3390/jcm13030744)
Supplement: Supplementary file 1 [file jcm-13-00744-s001.zip › jcm-2803299-supplementary.pdf]

OCENA JAKOŚCI FUNKCJONOWANIA PSYCHOFIZYCZNEGO DZIECI I MŁODZIEŻY W DOBIE PANDEMII COVID-19 W ZALEŻNOŚCI OD MIEJSCA ZAMIESZKANIA

Witajcie!
Ankieta, o której wypełnienie prosimy, skierowana jest do dzieci i młodzieży w wieku szkolnym (6-18 lat) i jest całkowicie anonimowa. Jej wyniki będą wykorzystane do opracowania naukowego. Tematy poruszane w ankiecie są dla nas psychiatrów bardzo ważne, sam pomysł stworzenia tego badania powstał w wyniku wielu rozmów z Waszymi koleżankami i kolegami, którzy prosili, aby zwrócić uwagę na ich problemy wynikające z izolacji w okresie pandemii COVID-19. Mamy świadomość, że jest to temat sprawiający wiele problemów, które my - dorośli nie zawsze wiemy jak rozwiązać. Wyniki ankiety oprócz wniosków związanych z pandemią mogą bardzo przydać się w przyszłości, pomóc rozumieć Was lepiej. Dlatego bardzo prosimy o wypełnienie poniższej ankiety. Zajmie to około 10 minut, a wyniki jakie uzyskamy mogą pozwolić dorosłym lepiej wspierać młodsze pokolenia w sytuacjach kryzysowych. Jeśli zechcecie pomóc w tym projekcie prosimy również o zachęcenie znajomych do wypełnienia ankiety. Im więcej informacji uzyskamy, tym lepiej.
Serdecznie dziękujemy i pozdrawiamy! :)

lek. Jagoda Grzejszczak, Klinika Psychiatrii Młodzieżowej CSK UM Łódź
prof. dr hab. n. med. Dominik Strzelecki, Klinika Zaburzeń Afektywnych i Psychotycznych CSK UM Łódź

Jeżeli jako Rodzic zgadzasz się na wzięcie udziału Twojego Dziecka w Naszych badaniach ankietowych udostępnij pytania Dziecku. Jeśli jesteś Uczniem, który otrzymał poniższy arkusz z pytaniami od Rodzica i zgadzasz się na udział w Naszych badaniach ankietowych przejdź prosimy do wypełniania poniższego kwestionariusza.

* Wskazuje wymagane pytanie

1. Płeć *

Zaznacz wszystkie właściwe odpowiedzi.

- [ ] mężczyzna (chłopak)
- [ ] kobieta (dziewczyna)

2. Data urodzenia (rok, np. 2005) *

____

3. W jakim miejscu mieszkałeś podczas pandemii? *

Zaznacz tylko jedną odpowiedź.

- Warszawa (stolica)
- miasto (od 100 tys. do 1 mln mieszkańców)
- miasto (od 20 tys. do 100 tys. mieszkańców)
- miasteczko (od 10 tys. do 20 tys. mieszkańców)
- miasteczko (poniżej 10 tys. mieszkańców)
- wieś

4. Gdzie mieszkałeś podczas pandemii? *

Zaznacz tylko jedną odpowiedź.

- blok mieszkalny
- dom szeregowy
- dom wolnostojący
- placówka wychowawcza/oświatowa (np.internat)

5. Czy masz swój pokój? *

Zaznacz tylko jedną odpowiedź.

- tak
- tak, ale dzielę go z kimś (np.rodzeństwo)
- nie

6. Czy w okresie pandemii miałeś swobodny dostęp do komputera, żeby móc swobodnie się komunikować czy uczestniczyć w zajęciach szkolnych? *

Zaznacz tylko jedną odpowiedź.

- tak
- nie

7. Ile osób zamieszkiwało z Tobą w domu w okresie pandemii (włączając Ciebie)? *

Zaznacz tylko jedną odpowiedź.

- 2
- 3
- 4
- 5
- 6 i więcej

8. Ile masz rodzeństwa? *

Zaznacz tylko jedną odpowiedź.

- 0
- 1
- 2
- 3
- 4 i więcej

9. Wykształcenie - na jakim etapie kształcenia jesteś? *

Zaznacz tylko jedną odpowiedź.

- szkoła podstawowa
- liceum ogólnokształcące
- liceum profilowane
- technikum
- zasadnicza szkoła zawodowa

10. Jakie zajęcia szkolne odbywały się w Twojej placówce w okresie pandemii (wybierz odpowiedź pasującą do >50% zajęć)? *

Zaznacz tylko jedną odpowiedź.

- stacjonarne
- zdalne
- hybrydowe

11. Jak oceniasz przygotowanie nauczycieli oraz możliwości techniczne szkoły do prowadzenia zajęć dydaktycznych w dobie pandemii COVID? *

Zaznacz tylko jedną odpowiedź.

	1	2	3	4	5	
źle						dobrze

12. Jak oceniasz chęć pomocy oraz empatyczne podejście nauczycieli w dobie pandemii COVID? *

Zaznacz tylko jedną odpowiedź.

	1	2	3	4	5	
źle						dobrze

13. Czy Twoim zdaniem nauczyciele w dobie pandemii COVID byli bardziej wyrozumiali w stosunku do uczniów niż przed pandemią? (3-bez zmian) *

Zaznacz tylko jedną odpowiedź.

	1	2	3	4	5	
mni	◯	◯	◯	◯	◯	bardziej

14. Czy ktoś z Twoich bliskich (rodzice, dziadkowie, rodzeństwo, rodzeństwo rodziców) jest zatrudniony w systemie ochrony zdrowia? *

Zaznacz tylko jedną odpowiedź.

- ◯ nie
- ◯ jako lekarz/-arka
- ◯ jako pielęgniarz/-arka
- ◯ inny pracownik medyczny mający w pracy kontakt z pacjentem

Ogólne samopoczucie w okresie pandemii

15. Jak określisz swój ogólny stan zdrowia PRZED pandemią w skali do 10? *

Zaznacz tylko jedną odpowiedź.

	1	2	3	4	5	6	7	8	9	10	
zły	◯	◯	◯	◯	◯	◯	◯	◯	◯	◯	doskonały

16. Jak określisz swój ogólny stan zdrowia W OKRESIE pandemii w skali do 10? *

Zaznacz tylko jedną odpowiedź.

	1	2	3	4	5	6	7	8	9	10	
zły	◯	◯	◯	◯	◯	◯	◯	◯	◯	◯	doskonały

17. Czy chorujesz przewlekle (cukrzyca, astma/alergia, choroby układu pokarmowego)? *

Zaznacz tylko jedną odpowiedź.

- tak
- nie

18. Jeśli tak - wpisz jakie to schorzenie, jeśli nie - wpisz "NIE" *

____

Stan psychiczny w okresie pandemii

19. Jak określisz swój psychiczny stan zdrowia PRZED pandemią w skali do 10? *

Zaznacz tylko jedną odpowiedź.

	1	2	3	4	5	6	7	8	9	10	
zły	◯	◯	◯	◯	◯	◯	◯	◯	◯	◯	doskonały

20. Jak określisz swój psychiczny stan zdrowia W OKRESIE pandemii w skali do 10? *

Zaznacz tylko jedną odpowiedź.

	1	2	3	4	5	6	7	8	9	10	
zły	◯	◯	◯	◯	◯	◯	◯	◯	◯	◯	doskonały

21. Czy PRZED okresem pandemii miałeś kłopoty ze zdrowiem psychicznym? (możliwych kilka odpowiedzi) *

Zaznacz wszystkie właściwe odpowiedzi.

- [ ] nie
- [ ] depresja
- [ ] zaburzenia lękowe
- [ ] zachowania impulsywne (np.samookaleczenia)
- [ ] myśli i tendencje samobójcze
- [ ] psychoza (np.schizofrenia)
- [ ] zaburzenia tikowe
- [ ] uzależnienie od alkoholu/innych SPA (substancji psychoaktywnych)

22. Czy PRZED okresem pandemii miałeś kłopoty ze zdrowiem psychicznym, co wiązało się z korzystaniem z porad specjalistów (psycholog, psychoterapeuta, psychiatra)? (możliwych kilka odpowiedzi) *

Zaznacz wszystkie właściwe odpowiedzi.

- [ ] nie
- [ ] depresja
- [ ] zaburzenia lękowe
- [ ] zachowania impulsywne (np.samookaleczenia)
- [ ] myśli i tendencje samobójcze
- [ ] psychoza (np.schizofrenia)
- [ ] zaburzenia tikowe
- [ ] uzależnienie od alkoholu/innych SPA (substancji psychoaktywnych)

23. Czy PRZED pandemią byłeś hospitalizowany z przyczyn psychiatrycznych? *

Zaznacz tylko jedną odpowiedź.

- ( ) tak
- ( ) nie

24. Czy W OKRESIE pandemii miałeś/masz kłopoty ze zdrowiem psychicznym (możliwych kilka odpowiedzi) *

Zaznacz wszystkie właściwe odpowiedzi.

- [ ] nie
- [ ] depresja
- [ ] zaburzenia lękowe
- [ ] zachowania impulsywne (np.samookaleczenia)
- [ ] myśli i tendencje samobójcze
- [ ] psychoza (np.schizofrenia)
- [ ] zaburzenia tikowe
- [ ] uzależnienie od alkoholu/innych SPA(substancji psychoaktywnych)

25. Czy W OKRESIE pandemii miałeś/masz kłopoty ze zdrowiem psychicznym, co wiązało się z korzystaniem z porad specjalistów (psycholog, psychoterapeuta, psychiatra)?(możliwych kilka odpowiedzi) *

Zaznacz wszystkie właściwe odpowiedzi.

- [ ] nie
- [ ] depresja
- [ ] zaburzenia lękowe
- [ ] zachowania impulsywne (np.samookaleczenia)
- [ ] myśli i tendencje samobójcze
- [ ] psychoza (np.schizofrenia)
- [ ] zaburzenia tikowe
- [ ] uzależnienie od alkoholu/innych SPA(substancji psychoaktywnych)

26. Czy W OKRESIE pandemii byłeś hospitalizowany z przyczyn psychiatrycznych? *

Zaznacz tylko jedną odpowiedź.

- ( ) tak
- ( ) nie

27. Czy OBECNIE jesteś w trakcie leczenia z uwagi na swój stan psychiczny? (możliwych kilka odpowiedzi) *

Zaznacz wszystkie właściwe odpowiedzi.

- [ ] nie
- [ ] psychoterapia
- [ ] leki przeciwdepresyjne (np. sertralina, fluoksetyna)
- [ ] leki uspokajające (np. ziołowe preparaty bez recepty, hydroksyzyna)
- [ ] leki przeciwpsychotyczne (np. arypiprazol, haloperydol)

Występowanie obaw związanych z zakażeniem COVID-19

28. Czy obawiałeś się, że zarazisz się koronawirusem, a jeśli tak to jak bardzo? *

Zaznacz tylko jedną odpowiedź.

	1	2	3	4	5	
wca	◯	◯	◯	◯	◯	w bardzo dużym stopniu

29. Czy obawiałeś się, że któryś z członków Twojej rodziny ulegnie zakażeniu COVID-19? *

Zaznacz tylko jedną odpowiedź.

	1	2	3	4	5	
wca	◯	◯	◯	◯	◯	w bardzo dużym stopniu

Potrzeba utrzymywania kontaktów społecznych w okresie pandemii

30. Czy odczuwałeś/-aś potrzebę komunikowania się/ kontaktu z innymi członkami rodziny w okresie pandemii? Jak oceniasz ją w stosunku do okresu przed pandemią? (3-bez zmian) *

Zaznacz tylko jedną odpowiedź.

	1	2	3	4	5	
mni	◯	◯	◯	◯	◯	większa

31. Czy odczuwałeś/-aś potrzebę komunikowania się/ kontaktu z innymi bliskimi Ci osobami (przyjaciele, koledzy) w okresie pandemii? Jak oceniasz ją w stosunku do okresu przed pandemią? (3-bez zmian) *

Zaznacz tylko jedną odpowiedź.

	1	2	3	4	5	
mni	◯	◯	◯	◯	◯	większa

32. Czy potrzebowałeś/-aś emocjonalnego wsparcia w okresie pandemii od członków rodziny? *

Zaznacz tylko jedną odpowiedź.

	1	2	3	4	5	
zna	◯	◯	◯	◯	◯	znacznie bardziej

33. Czy potrzebowałeś/-aś emocjonalnego wsparcia w okresie pandemii od przyjaciół, kolegów? *

Zaznacz tylko jedną odpowiedź.

	1	2	3	4	5	
zna	◯	◯	◯	◯	◯	znacznie bardziej

34. Czy zdarzały Ci się konflikty z osobami bliskimi w okresie pandemii? Jak oceniasz ją w stosunku do okresu przed pandemią? (3-bez zmian) *

Zaznacz tylko jedną odpowiedź.

	1	2	3	4	5	
rzad	◯	◯	◯	◯	◯	częściej

35. Jak ocenisz jakość Twoich kontaktów z osobami bliskimi w czasie pandemii w stosunku do okresu przed? (3-bez zmian) *

Zaznacz tylko jedną odpowiedź.

	1	2	3	4	5	
leps	◯	◯	◯	◯	◯	gorsze

Zajęcia szkolne W OKRESIE pandemii

36. Czy zdarzało Ci się opuszczać zajęcia szkolne W OKRESIE pandemii z przyczyn nieusprawiedliwionych (wagary)? *

Zaznacz tylko jedną odpowiedź.

- ◯ nie
- ◯ tak

37. Jeśli wagarowałeś/-aś to jak często w stosunku do okresu sprzed pandemii? (3-bez zmian) *

Zaznacz tylko jedną odpowiedź.

	1	2	3	4	5	
rzad	◯	◯	◯	◯	◯	częściej

38. Czy miałeś/-aś trudności w skupieniu na zajęciach w stosunku do okresu sprzed pandemii? (3-bez zmian) *

Zaznacz tylko jedną odpowiedź.

	1	2	3	4	5	
mni	◯	◯	◯	◯	◯	większe

39. Czy Twoje wyniki w nauce (oceny) w stosunku do okresu sprzed pandemii pogorszyły się czy polepszyły? (3-bez zmian) *

Zaznacz tylko jedną odpowiedź.

	1	2	3	4	5	
pole	◯	◯	◯	◯	◯	pogorszyły

Lęk i niepokój PRZED pandemią

PRZED pandemią...

40. Czułem/-am się bezpieczny/-a *

Zaznacz tylko jedną odpowiedź.

	1	2	3	4	5	
barc	◯	◯	◯	◯	◯	wcale

41. Czułem/-am się pełen/-a obaw *

Zaznacz tylko jedną odpowiedź.

	1	2	3	4	5	
wca	◯	◯	◯	◯	◯	bardzo

42. Czułem/-am się obciążony/-a *

Zaznacz tylko jedną odpowiedź.

	1	2	3	4	5	
wca	◯	◯	◯	◯	◯	bardzo

43. Czułem/-am się zdenerwowany/-a *

Zaznacz tylko jedną odpowiedź.

	1	2	3	4	5	
wca	◯	◯	◯	◯	◯	bardzo

44. Czułem/-am się rozdrażniony/-a *

Zaznacz tylko jedną odpowiedź.

	1	2	3	4	5	
abs(	◯	◯	◯	◯	◯	absolutnie tak

45. Często się martwiłem/-am *

Zaznacz tylko jedną odpowiedź.

	1	2	3	4	5	
abs(	◯	◯	◯	◯	◯	absolutnie tak

46. Czułem się zdezorientowany/-a (nie wiedziałeś/-aś co masz robić) *

Zaznacz tylko jedną odpowiedź.

	1	2	3	4	5	
abs(	◯	◯	◯	◯	◯	absolutnie tak

Lęk i niepokój W OKRESIE pandemii

W OKRESIE pandemii...

47. Czułem/-am się bezpieczny/-a *

Zaznacz tylko jedną odpowiedź.

	1	2	3	4	5	
barc	◯	◯	◯	◯	◯	wcale

48. Czułem/-am się pełen/-a obaw *

Zaznacz tylko jedną odpowiedź.

	1	2	3	4	5	
wca	◯	◯	◯	◯	◯	bardzo

49. Czułem/-am się obciążony/-a *

Zaznacz tylko jedną odpowiedź.

	1	2	3	4	5	
wca	◯	◯	◯	◯	◯	bardzo

50. Czułem/-am się zdenerwowany/-a *

Zaznacz tylko jedną odpowiedź.

	1	2	3	4	5	
wca	◯	◯	◯	◯	◯	bardzo

51. Czułem/-am się rozdrażniony/-a *

Zaznacz tylko jedną odpowiedź.

	1	2	3	4	5	
abs(	◯	◯	◯	◯	◯	absolutnie tak

52. Często się martwiłem/-am *

Zaznacz tylko jedną odpowiedź.

	1	2	3	4	5	
abs(	◯	◯	◯	◯	◯	absolutnie tak

53. Czułem się zdezorientowany/-a (nie wiedziałeś/-aś co masz robić) *

Zaznacz tylko jedną odpowiedź.

	1	2	3	4	5	
abs(	◯	◯	◯	◯	◯	absolutnie tak

54. Jak bardzo zmienił się Twój stan emocjonalny związany z powyższymi odczuciami w stosunku do okresu sprzed pandemii (3-bez zmian)? *

Zaznacz tylko jedną odpowiedź.

	1	2	3	4	5	
znac	◯	◯	◯	◯	◯	znacznie się pogorszył

Objawy depresyjne PRZED pandemią

PRZED pandemią...

55. Czułem/-am się bezwartościowy *

Zaznacz tylko jedną odpowiedź.

	1	2	3	4	5	
wca	◯	◯	◯	◯	◯	bardzo

56. Czułem/-am, że moje problemy są nierozwiązywalne *

Zaznacz tylko jedną odpowiedź.

	1	2	3	4	5	
abs(	◯	◯	◯	◯	◯	absolutnie tak

57. Czułem/-am, że świat nie ma sensu *

Zaznacz tylko jedną odpowiedź.

	1	2	3	4	5	
abs(	◯	◯	◯	◯	◯	absolutnie tak

58. Miałem/-am kłopoty z apetytem *

Zaznacz tylko jedną odpowiedź.

	1	2	3	4	5	
wca	◯	◯	◯	◯	◯	bardzo

59. Brakowało mi energii do wykonywania codziennych czynności *

Zaznacz tylko jedną odpowiedź.

	1	2	3	4	5	
wca	◯	◯	◯	◯	◯	bardzo

60. To co wcześniej sprawiało mi radość - przestało mnie cieszyć *

Zaznacz tylko jedną odpowiedź.

	1	2	3	4	5	
abs(	◯	◯	◯	◯	◯	absolutnie tak

61. Miałem/-am problemy ze snem (trudności w zaśnięciu, częste wybudzenia w nocy, koszmary senne) *

Zaznacz tylko jedną odpowiedź.

	1	2	3	4	5	
abs(	◯	◯	◯	◯	◯	absolutnie tak

62. Czułem/-am się samotny/-a *

Zaznacz tylko jedną odpowiedź.

	1	2	3	4	5	
abs(	◯	◯	◯	◯	◯	absolutnie tak

63. Często płakałem/-am *

Zaznacz tylko jedną odpowiedź.

	1	2	3	4	5	
abs(	◯	◯	◯	◯	◯	absolutnie tak

Objawy depresyjne W OKRESIE pandemii

W OKRESIE pandemii...

64. Czułem/-am się bezwartościowy *

Zaznacz tylko jedną odpowiedź.

	1	2	3	4	5	
wca	◯	◯	◯	◯	◯	bardzo

65. Czułem/-am, że moje problemy są nierozwiązywalne *

Zaznacz tylko jedną odpowiedź.

	1	2	3	4	5	
abs(	◯	◯	◯	◯	◯	absolutnie tak

66. Czułem, że świat nie ma sensu *

Zaznacz tylko jedną odpowiedź.

	1	2	3	4	5	
abs(	◯	◯	◯	◯	◯	absolutnie tak

67. Miałem/-am kłopoty z apetytem *

Zaznacz tylko jedną odpowiedź.

	1	2	3	4	5	
wca	◯	◯	◯	◯	◯	bardzo

68. Brakowało mi energii do wykonywania codziennych czynności *

Zaznacz tylko jedną odpowiedź.

	1	2	3	4	5	
wca	◯	◯	◯	◯	◯	bardzo

69. To co wcześniej sprawiało mi radość - przestało mnie cieszyć *

Zaznacz tylko jedną odpowiedź.

	1	2	3	4	5	
abs(	◯	◯	◯	◯	◯	absolutnie tak

70. Miałem/-am problemy ze snem (trudności w zaśnięciu, częste wybudzenia w nocy, koszmary senne) *

Zaznacz tylko jedną odpowiedź.

	1	2	3	4	5	
abs(	◯	◯	◯	◯	◯	absolutnie tak

71. Czułem/-am się samotny/-a *

Zaznacz tylko jedną odpowiedź.

	1	2	3	4	5	
abs(	◯	◯	◯	◯	◯	absolutnie tak

72. Często płakałem/-am *

Zaznacz tylko jedną odpowiedź.

	1	2	3	4	5	
abs(	◯	◯	◯	◯	◯	absolutnie tak

73. Jak bardzo zmienił się Twój stan emocjonalny związany z powyższymi odczuciami w stosunku do okresu sprzed pandemii (3-bez zmian) *

Zaznacz tylko jedną odpowiedź.

	1	2	3	4	5	
zna(	◯	◯	◯	◯	◯	znacznie się pogorszył

Zachowania agresywne i autoagresywne

74. Czy PRZED pandemią dokonywałeś samookaleczeń? *

Zaznacz tylko jedną odpowiedź.

- nie
- tak

75. Jeśli zaznaczyłeś/-aś "tak" - co dawały Ci samookaleczenia PRZED pandemią?

Zaznacz wszystkie właściwe odpowiedzi.

- poczucie ulgi, redukcję napięcia
- radość, euforię
- robię to, gdy się nudzę
- traktuję to jako sposób na śmierć

76. Czy W OKRESIE pandemii dokonywałeś samookaleczeń? *

Zaznacz tylko jedną odpowiedź.

- nie
- tak

77. Jeśli zaznaczyłeś/-aś "tak" - co dawały Ci samookaleczenia W OKRESIE pandemii?

Zaznacz wszystkie właściwe odpowiedzi.

- poczucie ulgi, redukcję napięcia
- radość, euforię
- robię to, gdy się nudzę
- traktuję to jako sposób na śmierć

78. Czy zdarzało Ci się kogoś uderzyć/wdać w bójkę PRZED pandemią? *

Zaznacz tylko jedną odpowiedź.

- nie
- tak

79. Czy zdarzało Ci się kogoś uderzyć/wdać w bójkę W OKRESIE pandemii? *

Zaznacz tylko jedną odpowiedź.

- nie
- tak

80. Czy byłeś ofiarą przemocy fizycznej PRZED pandemią? *

Zaznacz tylko jedną odpowiedź.

- nie
- tak

81. Czy byłeś ofiarą przemocy fizycznej W OKRESIE pandemii? *

Zaznacz tylko jedną odpowiedź.

- nie
- tak

82. Czy byłeś ofiarą przemocy psychicznej PRZED pandemią? *

Zaznacz tylko jedną odpowiedź.

- nie
- tak

83. Czy byłeś ofiarą przemocy psychicznej W OKRESIE pandemii? *

Zaznacz tylko jedną odpowiedź.

- nie
- tak

84. Jak zmieniło się Twoje zachowanie w zakresie agresji i autoagresji w stosunku do okresu sprzed pandemii (3-bez zmian)? *

Zaznacz tylko jedną odpowiedź.

	1	2	3	4	5	
zach	◯	◯	◯	◯	◯	zach[illegible]esywnych jest znacznie więcej

85. Jak często podczas pandemii byłeś ofiarą przemocy psychicznej/fizycznej w stosunku do okresu sprzed pandemii? (3-bez zmian) *

Zaznacz tylko jedną odpowiedź.

	1	2	3	4	5	
wca	◯	◯	◯	◯	◯	częściej

Myślenie o śmierci/ myśli i tendencje suicydalne

86. Czy myślałeś/-aś o śmierci PRZED pandemią? *

Zaznacz tylko jedną odpowiedź.

	1	2	3	4	5	
wca	◯	◯	◯	◯	◯	często

87. Czy PRZED pandemią występowały u Ciebie myśli samobójcze? *

Zaznacz tylko jedną odpowiedź.

	1	2	3	4	5	
wca	◯	◯	◯	◯	◯	często

88. Jeśli "tak" - czy myślałeś/-aś o sposobach na samobójstwo PRZED pandemią?

Zaznacz tylko jedną odpowiedź.

	1	2	3	4	5	
wca	◯	◯	◯	◯	◯	często

89. Czy PRZED pandemią podjąłeś/aś próbę samobójczą? *

Zaznacz tylko jedną odpowiedź.

- ◯ nie
- ◯ tak - jeden raz
- ◯ tak - kilkukrotnie

90. Jak często myślałeś/-aś o śmierci W OKRESIE pandemii? *

Zaznacz tylko jedną odpowiedź.

	1	2	3	4	5	
wca	◯	◯	◯	◯	◯	często

91. Czy W OKRESIE pandemii występowały u Ciebie myśli samobójcze? *

Zaznacz tylko jedną odpowiedź.

	1	2	3	4	5	
wca	◯	◯	◯	◯	◯	często

92. Jeśli "tak" - czy myślałeś/-aś o sposobach na samobójstwo W OKRESIE pandemii?

Zaznacz tylko jedną odpowiedź.

	1	2	3	4	5	
wca	◯	◯	◯	◯	◯	często

93. Czy W OKRESIE pandemii podjąłeś/aś próbę samobójczą? *

Zaznacz tylko jedną odpowiedź.

- nie
- tak - jeden raz
- tak - kilkukrotnie

Substancje psychoaktywne W OKRESIE pandemii

94. palenie tytoniu przed pandemią *

Zaznacz tylko jedną odpowiedź.

- nie paliłem
- paliłem okazjonalnie
- paliłem regularnie

95. spożywanie alkoholu przed pandemią *

Zaznacz tylko jedną odpowiedź.

- nie piłem
- piłem okazjonalnie
- piłem regularnie

96. inne używki przed pandemią (marihuana, amfetamina, dopalacze, inne) *

Zaznacz tylko jedną odpowiedź.

- nie zażywałem
- zażywałem okazjonalnie
- zażywałem regularnie

97. w porównaniu do okresu wcześniejszego - w okresie pandemii paliłeś/-aś (3-bez zmian) *

Zaznacz tylko jedną odpowiedź.

	1	2	3	4	5	
rzad	○	○	○	○	○	częściej

98. w porównaniu do okresu wcześniejszego - w okresie pandemii spożywałeś/-aś alkohol (3-bez zmian) *

Zaznacz tylko jedną odpowiedź.

	1	2	3	4	5	
rzad	○	○	○	○	○	częściej

99. w porównaniu do okresu wcześniejszego - w okresie pandemii sięgałeś/-aś po inne używki (3-bez zmian) *

Zaznacz tylko jedną odpowiedź.

	1	2	3	4	5	
rzad	○	○	○	○	○	częściej

Aktywność fizyczna W OKRESIE pandemii

100. Czy uprawiasz regularnie jakiś sport? (2-3 razy w tygodniu) *

Zaznacz tylko jedną odpowiedź.

- nie
- tak

101. Czy i jak zmieniła się częstotliwość uprawianego przez Ciebie sportu W OKRESIE pandemii w stosunku do okresu przed nią? (zaznacz odpowiedź jeśli na poprzednie pytanie odpowiedziałeś twierdząco)

Zaznacz tylko jedną odpowiedź.

- częściej
- tak samo często
- rzadziej

102. Czy sport pomaga Ci w redukowaniu napięcia, odpręża, zmniejsza poziom lęku?

Zaznacz tylko jedną odpowiedź.

- bardzo
- trochę
- nie wpływa

Dziękujemy za wypełnienie ankiety

Ta treść nie została utworzona ani zatwierdzona przez Google.

Formularze Google
